# miR‐138‐5p targets MCU to inhibit mitochondrial biogenesis and colorectal cancer growth

**DOI:** 10.1111/jcmm.17798

**Published:** 2023-06-01

**Authors:** Jianjun Zhu, Chunle Zhang, Zhengjie Wang, Lihong Shi, Li Li, Hao Wu, Ming Liu

**Affiliations:** ^1^ Department of Medical Cellular Biology and Genetics Shanxi Medical University Taiyuan China; ^2^ Department of Nephrology, Kidney Research Institute West China Hospital of Sichuan University Chengdu China; ^3^ Department of Nuclear Medicine The First Affiliated Hospital of Chongqing Medical University Chongqing China; ^4^ Department of Human Anatomy Shanxi Medical University Taiyuan China

**Keywords:** colorectal carcinoma, MCU, miR‐138‐5p, ROS, tumour growth

## Abstract

miR‐138‐5p has been identified as a novel cancer‐related miRNA molecule in a variety of malignancies. However, the functions and mechanisms underlying miR‐138‐5p in colorectal carcinoma (CRC) remains largely unknown. In the present study, we analysed the biological effects and clinical significance of miR‐138‐5p in CRC. miR‐138‐5p expression was analysed by quantitative real‐time PCR in CRC tissues and cell lines. The effects of miR‐138‐5p on CRC cell growth was detected by cell proliferation, colony formation, cell cycle and cell apoptosis assays in vitro and in vivo. Our data showed that miR‐138‐5p was significantly downregulated in CRC. Downregulated miR‐138‐5p was related with poor prognosis in patients with CRC. miR‐138‐5p suppressed CRC growth but promoted cell death both in vitro and in vivo. Online predictions and integrated experiments identified that miR‐138‐5p targeted MCU, and downregulated miR‐138‐5p promoted mitochondrial biogenesis in CRC. In the light of the underlying mechanisms, our results indicated that downregulated miR‐138‐5p led to increased expression of MCU, which subsequently increased the production of ROS to promote CRC growth. Our results indicated that downregulated miR‐138‐5p strengthened mitochondrial biogenesis through targeting MCU, thus contributing to CRC cell growth, which may provide a potential therapeutic target for CRC.

## 1 INTRODUCTION

More than 1.9 million new colorectal cancer (CRC) cases and 935,000 deaths were estimated to occur in 2020.^1^ Globally, CRC ranks third in the light of incidence, but second in the light of mortality.^1^ There has been great progress in the diagnosis and treatment for CRC, but the 5‐year overall survival rate of patients with CRC remains low due to tumour recurrence, distant metastasis and resistance to chemoradiotherapy.^1^, ^2^ Although cancer has been studied over the past few decades through oncogenes and cancer suppressor genes, current understanding on its molecular pathogenesis are still insufficient to win the battle.^2^ Thus, studies that explore the molecular pathogenesis for the malignancy growth of CRC are urgently needed to develop novel treatment strategies.

MicroRNAs (miRNAs), approximately 23 nucleotides in length, are the smallest member of noncoding RNA molecules which function important roles in regulating the expression of protein‐coding genes.^2^ So far, approximately 60% of human coding genes has been claimed to be regulated by miRNAs.^2^ Moreover, miRNAs in cancer cells has been shown to be involved in a variety of different signalling pathways, including cell proliferation and apoptosis, differentiation, cell cycle, distant metastasis, cell metabolism and chemotherapy resistance.^2^, ^3^ In addition, aberrant expression of certain miRNAs has been proved to be a promising druggable target in cancers due to the fact that a single miRNA might revert the therapeutically unfavourable gene expression by targetting more than one gene.^2^

MiR‐138 was derived from two primary transcripts, pri‐miR‐138‐1 and pri‐miR‐138‐2.^2^ Several studies has reported that miR‐138‐5p is dysregulated in a variety of malignancies, including hepatocellular carcinoma,^4^ colorectal carcinoma,^3^, ^5^, ^6^ gastric cancer,^7^ pancreatic cancer,^8^, ^9^ ovarian cancer^10^ and oesophageal squamous cell carcinoma,^11^ which strongly suggested that miR‐138‐5p might function a tumour suppressive role in cancers. In recent years, several studies are beginning to focus on the biological role of miR‐138‐5p in CRC. Such a miR‐138 was reported to inhibit cell proliferation in colon cancer.^5^ Xu and colleagues demonstrated that miR‐138‐5p was downregulated in colorectal cancer, and miR‐138‐5p attenuated cell migration and was negatively related with resistance of chemoradiotherapy of colorectal cancer.^3^ Furthermore, Xu et al. reported that overexpression of miR‐138 suppressed cell growth and metastasis in CRC.^12^ However, the functions of miR‐138‐5p in the regulation of tumourigenesis and progression of CRC remains unclear. In order to elaborate the potential role of miR‐138‐5p in CRC growth, we systematically investigated the expression level of miR‐138‐5p and functional role of miR‐138‐5p in CRC.

## 2 MATERIALS AND METHODS

### 2.1 Microarray analysis

Detailed information of the microarray analysis was described in Data S1.

### 2.2 Cell culture and tissue collection

Human colon cell lines and human normal colorectal cell line FHC were cultured in DMEM medium supplemented with 10% FBS, and the detailed information was described in Data S1. A total of 30 pairs of tumour tissues and paired adjacent non‐tumour tissues were acquired from the Affiliated Hospital of Shanxi Medical University. Pathological examination further confirmed the absence of cancer cells in the normal collected tissues. This study gained the approval of the Ethical Committee of the Shanxi Medical University. All participants signed the informed consent.

### 2.3 miRNA mimics and miRNA inhibitor transduction

MiRNA mimics and miRNA inhibitor were designed ad synthesized by General Biology. The transfection of siRNAs or miRNA mimics/inhibitor was performed according to the manufacturer's instructions using Lipofectamine 2000 (Invitrogen). The sequences of miRNA mimics and miRNA inhibitors were listed in Table S1.

### 2.4 Cell proliferation assay

The cell proliferation assay was performed using the Cell Counting Kit (CCK‐8) and ethynyl deoxyuridine (EdU) incorporation assay kit (Ribobio). The CCK‐8 and EdU assays were performed according to the manufacturer's instructions, respectively.

### 2.5 Colony formation assay

To evaluate the colony formation assay, 500 cells were seeded into 25 cm^2^ cell culture plate. After 14 days of routine incubation, cells were washed with PBS buffer, and the colonies were methanol‐fixed and stained with crystal violet (1%).

### 2.6 Cell apoptosis and cell cycle analysis

Cell apoptosis assay was performed using the Annexin V‐FITC Kit (BestBio) according to the manufacturer's instructions. TUNEL assay was carried out as described in Data S1. The cell cycle analysis was performed using the cell cycle detection regent kit (BestBio) according to the manufacturer's instructions.

### 2.7 Nude mice xenograft model

Four‐week‐old male BALB/c nude mice were subcutaneously injected with 1 × 10^7^ Ls174T cells. Ten days later, AgomiR‐138 group mice were injected with AgomiR‐138 (10 nmol) every 10 days for two times via the tumour regional injection, respectively. The control group mice were injected with control AgomiR. The tumour volume (*V*, mm^3^) was calculated with the formula *V* = (length × width^2^)/2. Then 4 weeks later, the nude mice were sacrificed and tumours were removed for qRT‐PCR, and TUNEL assay. Animal study was approved by the Animal Care and Use Committee of Shanxi Medical University.

### 2.8 qPCR and western blotting

qPCR and western blot analysis were carried out as described in Data S1.The primer sequences used in the present study were shown in Table S2. Antibodies used in this study were listed in Table S3.

### 2.9 Dual luciferase reporter gene assay

The wide type (WT) sequence and the corresponding mutant type (MUT) sequence based on the binding site between MCU and miR‐138‐5p were inserted into the psiCheck2 vector to construct psiCheck2‐MCU‐WT vector and psiCheck2‐MCU‐MUT vector. Then, the psiCheck2‐MCU‐WT vector or psiCheck2‐MCU‐MUT vector was co‐transfected with miR‐138‐5p mimic or miR‐138‐5p inhibitor into CRC cells. The luciferase activity was determined according to the instruction of the dual luciferase reporter assay system (Promega).

### 2.10 Measurement of mitochondrial Ca^2+^

Cells were loaded with Rhod‐2/AM (Invitrogen) for 20 min at 37°C. The, cells were washed with PBS buffer to remove any dye, and then incubated for a further 30 min to allow complete de‐esterification of intracellular AM esters. Images were obtained with a laser confocal microscope FV1000 (Olympus).

### 2.11 Measurement of mitochondrial mass

A three‐dimensional (3D) model of mitochondrial was constructed using the method as previously described. Briefly, 100 nM Mitotracker Red was applied to label mitochondria, and warmed PBS buffer was used to wash the cells. An inverted laser confocal microscope with a high‐sensitivity CCD camera was employed to obtain images. A total of 15 image planes were obtained along the z‐axis at 0.2 μm increments. Three‐dimensional data processing and morphometric analysis were performed using the Imaris 7.1.1 software (Bitplane).

### 2.12 Measurement of mtDNA copy number

Total DNA was isolated from CRC cells using TIANamp Genomic DNA Kit (Tiangen) according to the manufacturer's instructions. A qPCR‐based method was employed to quantify the relative mtDNA copy number, as previously described.

### 2.13 Measurement of ATP production

For the ATP assay, cells cultured in 6‐well plates were lysed in 200 μL/well lysis buffer on ice and centrifuged at 4°C. The ATP content in the supernatant was determined using an enhanced ATP assay kit (Beyotime Biotechnology) according to the recommended protocol. The content was normalized to the cell number.

### 2.14 Measurement of reactive oxygen species

Cellular reactive oxygen species (ROS) were detected using the fluorescence probe DCF‐DA (Beyotime) according to the manufacturer's instructions.

### 2.15 Statistical analysis

All statistical analyses were performed using Prime v6 (GraphPad software Inc.). Data were presented as the mean ± SD from three independent experiments, where appropriate. Student's *t*‐test was carried out to analyse the significant difference between two groups. One‐way anova by Tukey's test was carried to analyse multiple comparisons. A paired two‐tailed *t‐*test was employed to compare the difference between tumour tissues ad paired adjacent non‐tumour tissues. Correlations were analysed by Pearson's or Spearman's test. The Kaplan–Meier method with the log‐rank test was used for survival analysis. *p* < 0.05 was considered statistically significant.

## 3 RESULTS

### 3.1 Downregulation of miR‐138‐5p is associated with poor prognosis in patients with CRC

**FIGURE 1:**
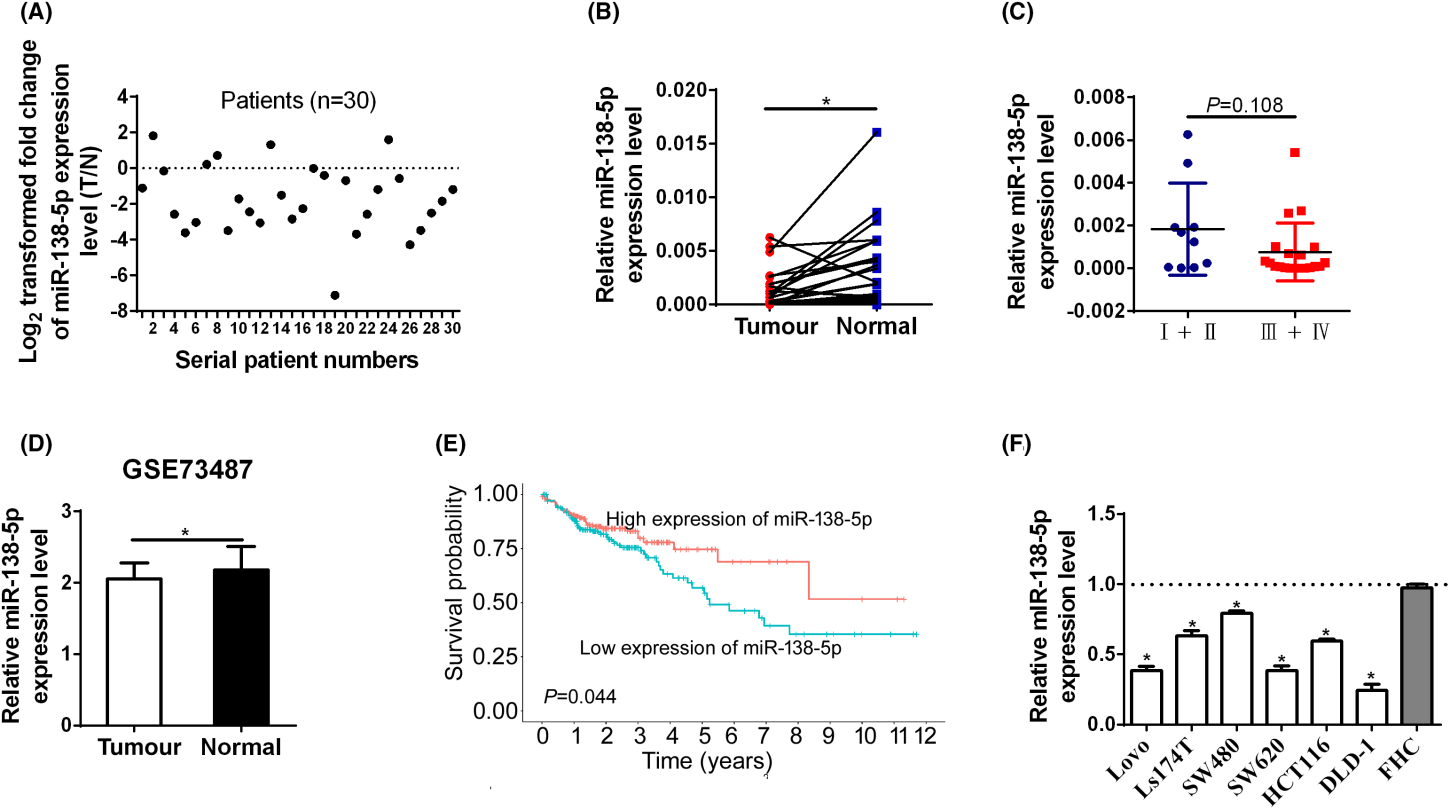
miR‐138‐5p was downregulated and associated with poor prognosis in patients with CRC. (A and B) miR‐138‐5p expression was analysed by qRT‐PCR in paired tumour (T) and normal (N) tissues (*n* = 30). (C) The expression of miR‐138‐5p was analysed in CRC tissues with early stage and advanced stage. (D) Bioinformatics analysis for miR‐138‐5p expression based on the GSE73487 dataset. (E) Kaplan–Meier survival curves for overall survival (OS) stratified by miR‐138‐5p expression in CRC patients. (F) miR‐138‐5p expression was analysed by qRT‐PCR in CRC cell lines and normal colon cell line. *p* values were shown as: *p < 0.05.

In order to determine the expression of miR‐138‐5p in CRC, we performed quantitative PCR (qPCR) assay on 30 paired CRC tissues and adjacent non‐tumour tissues and found that the expression of miR‐138‐5p was significantly lower in CRC tissues (Figure 1A,B). Moreover, the expression of miR‐138‐5p was gradually decreased with the tumour stage of CRC (Figure 1C). To further determine the expression pattern of miR‐138‐5p in CRC, we searched the GEO database, and the results indicated that the expression of miR‐138‐5p was lower in CRC tissues (Figure 1D). The above findings were verified again by the bioinformatics analysis based on other two GEO datasets, and the expression of miR‐138‐5p was lower in CRC tissues, but with no significant differences between tumour and adjacent non‐tumour tissues groups (*p* > 0.05; Figure S1A).

Additionally, CRC patients enrolled in our study were stratified into two groups based on the median expression level of miR‐138‐5p in CRC tissues. Kaplan–Meier analysis with log‐rank test presented that CRC patients that had a low expression of miR‐138‐5p displayed slower overall survival (OS) compared with those who had high expression of miR‐138‐5p (Figure 1E). Furthermore, survival analysis indicated that patients with low levels of miR‐138‐5p suffered poorer OS in the early stage group and in the advanced stage group, respectively, but both the differences did not reach statistical significance, which might be due to limitation in sample size (Figure S1B,C). The expression level of miR‐138‐5p in common CRC cell lines was also detected by qRT‐PCR, and our results showed that miR‐138‐5p was lowly expressed in all six CRC cell lines compared with the normal colonic cell line (Figure 1F). Collectively, these results indicated that miR‐138‐5p was downregulated in CRC tissues, and downregulated miR‐138‐5p was associated with poor prognosis in CRC patients.

### 3.2 miR‐138‐5p suppressed CRC cell growth in vitro

**FIGURE 2:**
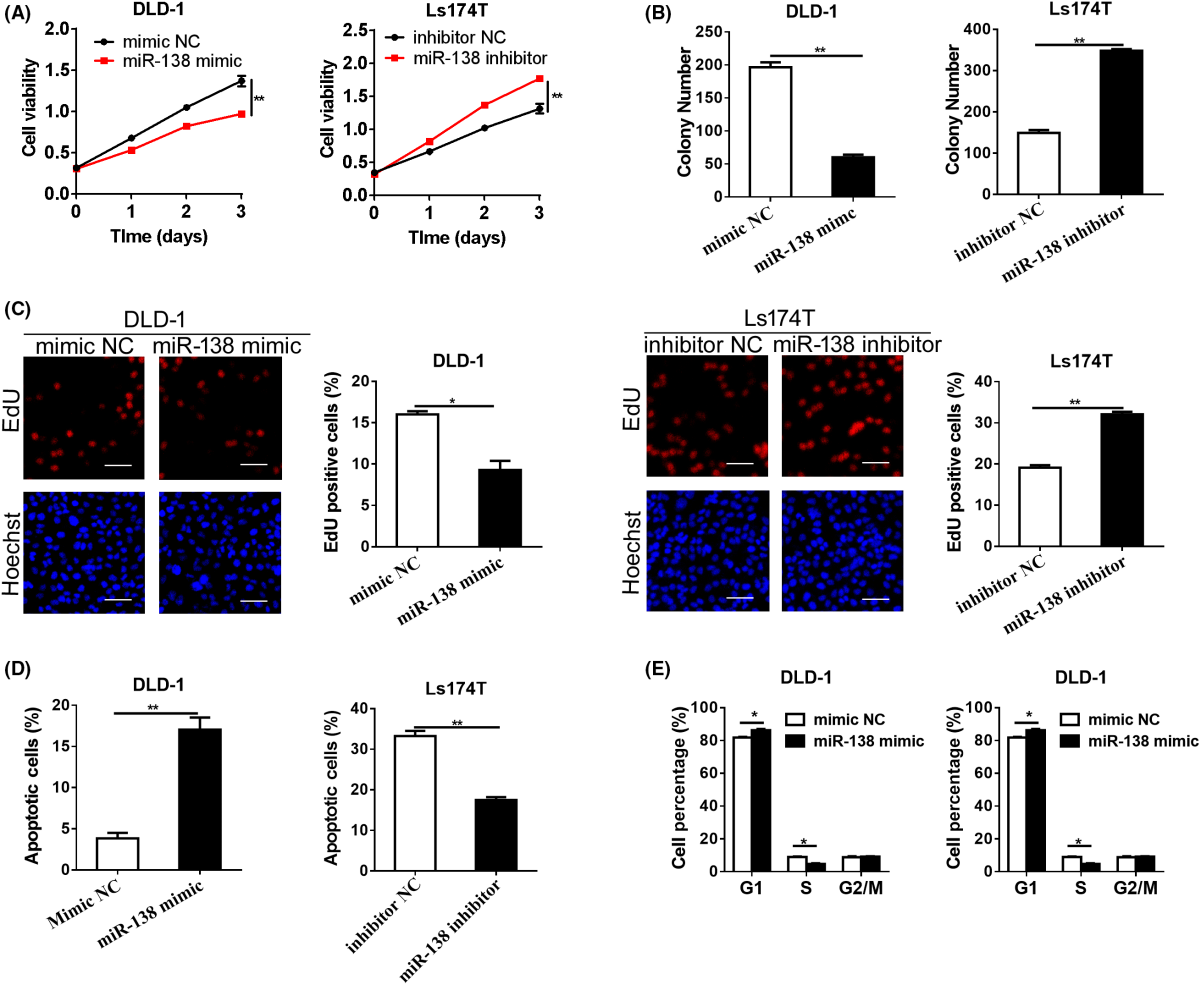
miR‐138‐5p inhibited CRC cell growth and induced apoptosis. (A and B) CCK‐8 and colony formation assays were applied for cell growth analysis in DLD‐1 and Ls174‐T cells (minic NC, control miRNA mimic; inhibitor NC, control miRNA inhibitor). (C) Cell proliferation ability was evaluated using ethynyl deoxyuridine (EdU) incorporation assay 48 h after transfection with treatment as indicated. Scale bar, 50 μm. (D) Flow cytometry analysis of cell apoptosis by Annexin‐V/PI staining in CRC cells, treated as indicated. 5‐FU, 5‐fluorouracil (50 μm/L). (E) Cell cycle analyses by flow cytometry in CRC cells 48 h after treatment as indicated. *p* values were shown as: *p < 0.05, **p < 0.01.

To determine the role of miR‐138‐5p in CRC cell growth, miR‐138‐5p mimic or miR‐138‐5p inhibitor was applied to upregulate or downregulate the level of miR‐138‐5p in CRC cells, respectively. As shown in Figures 2A and S2A, upregulation of miR‐138‐5p attenuated cell growth compared with the control group, whereas the contrary result was obtained when the expression of miR‐138‐5p was downregulated in CRC cells. Furthermore, these findings were supported by the colony formation assay and EdU incorporation assay (Figures 2B,C and S2B,C). We also explored the effect of miR‐138‐5p on cell apoptosis in CRC cells. Flow cytometric analysis indicated that upregulation of miR‐138‐5p notably promoted cell apoptosis compared with controls, while cell apoptosis induced by 5‐FU was remarkably suppressed when miR‐138‐5p expression was downregulated in CRC cells (Figures 2D and S2D).

It is well known that fast cell cycle progression accounts for cancer proliferation. To this end, flow cytometric analysis was applied to detect the cell cycle in CRC cells. As shown in Figure 2E, upregulation of miR‐138‐5p led to a notable cell cycle arrest at the G1 phase, whereas downregulation of miR‐138‐5p led to a decreased rate of G1 phase in CRC cells. Collectively, these results demonstrated that miR‐138‐5p suppressed G1‐S phase transition, which might contribute to the attenuated growth in CRC.

### 3.3 miR‐138‐5p suppressed tumour progression in CRC in vivo

**FIGURE 3:**
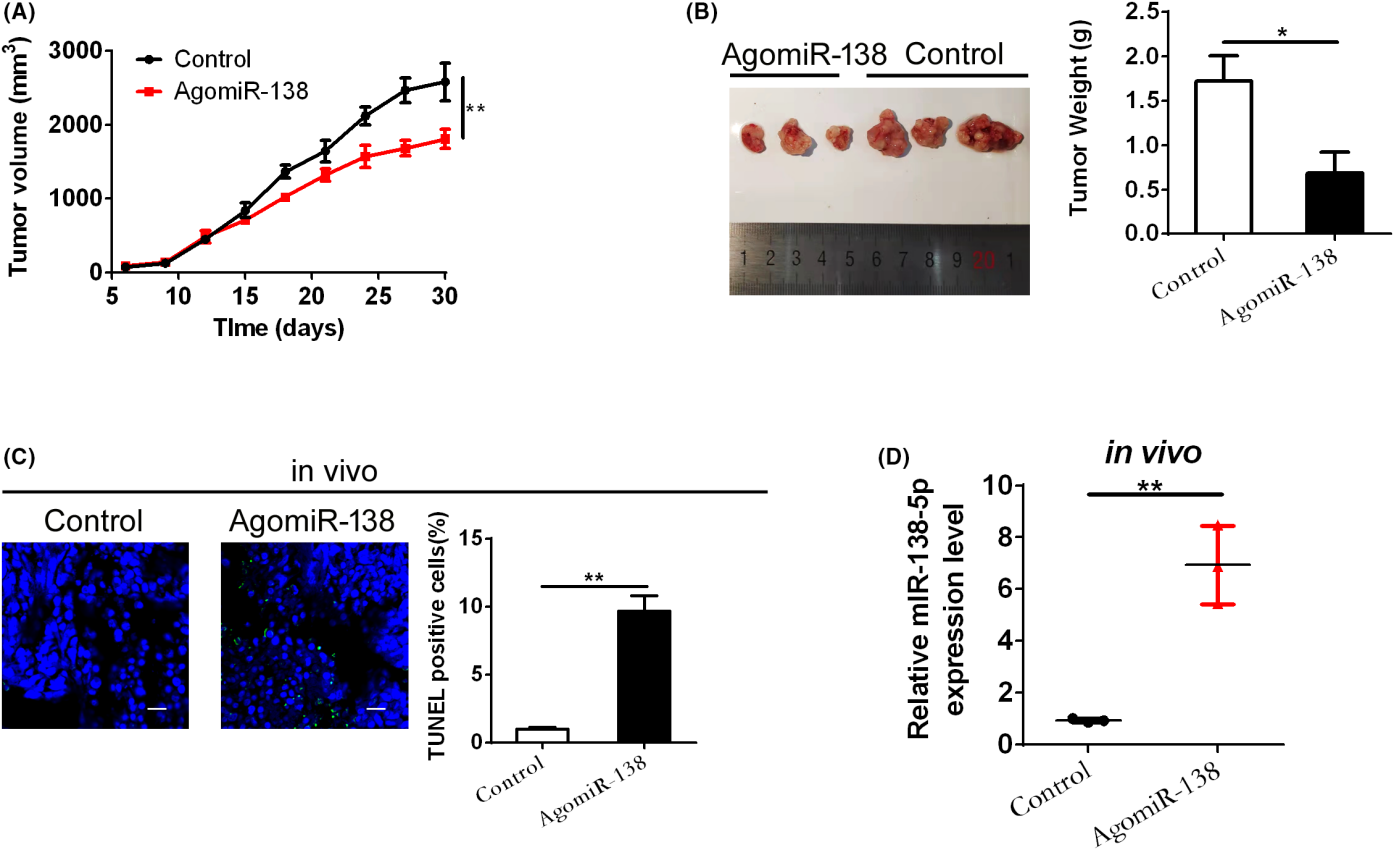
miR‐138‐5p suppressed CRC growth in vivo. (A) Tumour growth curves of subcutaneous xenograft tumour developed from Ls174T cells, treated as indicated. (B) Dissected tumours from sacrificed mice and tumour weight were calculated and shown. (C) TUNEL staining in tumour tissues of subcutaneous xenografts with the indicated treatments. Blue: Hoechst 33342; Green:TUNEL positive nucleus; Scale bar, 50 μm. (D) miR‐138‐5p expression was analysed by qPCR in subcutaneous xenograft tumours. *p* values were shown as: *p < 0.05, **p < 0.01.

Based on the above findings, miR‐138‐5p was speculated to play a vital role in tumour growth in vivo. The effect of miR‐138‐5p on tumour growth was studied in vivo by generating a CRC xenograft nude mice model. As shown in Figure 3A, the growth rate of xenografts with miR‐138‐5p agomir treatment was slower compared with the controls. The tumour weight was significantly lighter in the miR‐138‐agomir group compared with the controls (Figure 3B). Furthermore, we confirmed the effect of miR‐138‐5p on apoptosis in xenograft nude mice models. CRC xenografts with miR‐138‐5p agomir treatment presented a notable increase in TUNEL staining compared with the corresponding controls (Figure 3C). To confirm whether the expression level of miR‐138‐5p was upregulated in the miR‐138‐5p agomir group, qPCR assay was performed to detect the expression of miR‐138‐5p. As shown in Figure 3D, the expression of miR‐18‐5p was significantly increased in the miR‐138‐5p agomir group compared with the controls. Taken together, these results indicated that miR‐138‐5p suppressed CRC progression in vivo.

### 3.4 MCU was the potential target of miR‐138‐5p in CRC

**FIGURE 4:**
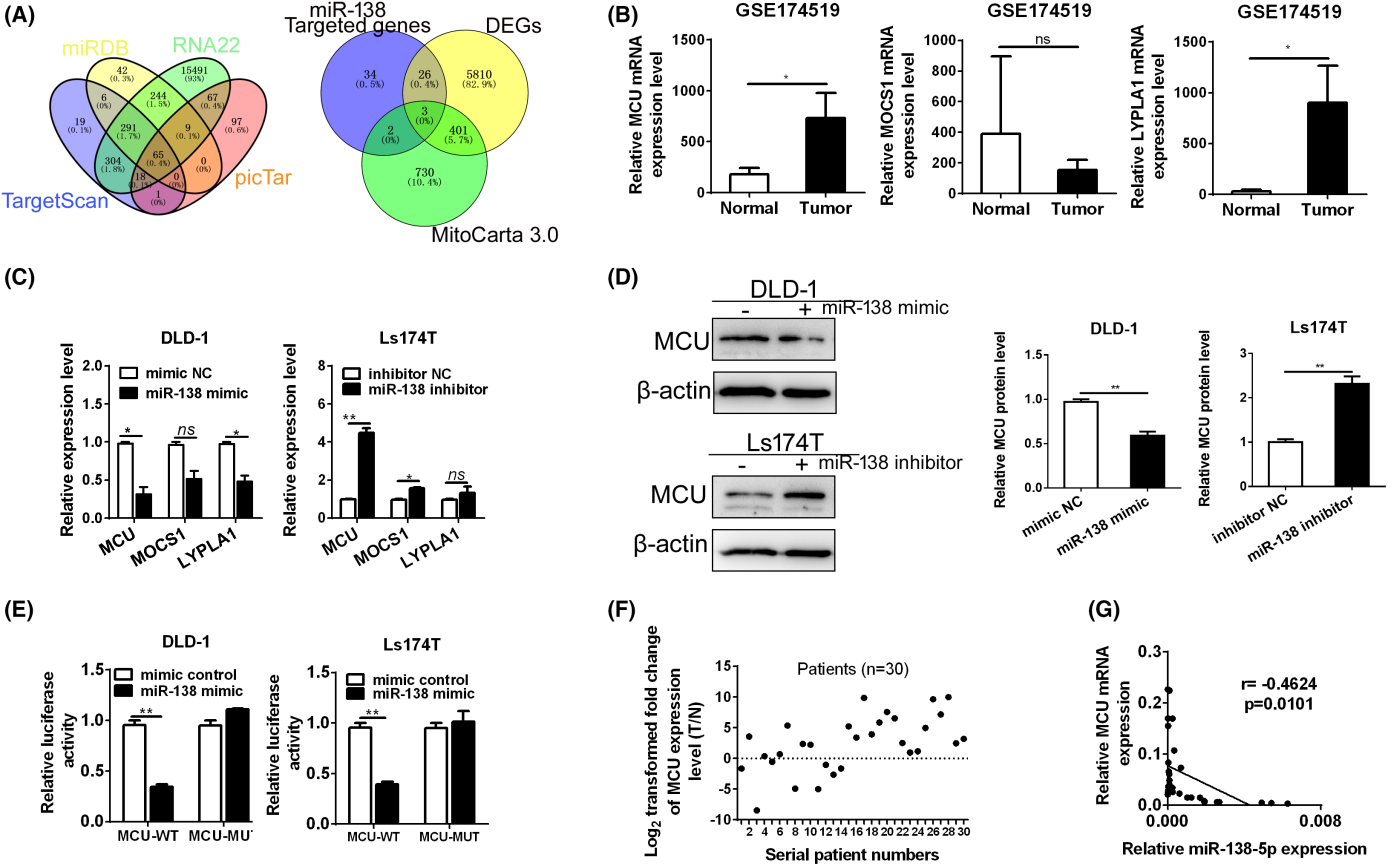
miR‐138‐5p could target MCU (A) Venn diagram of the intersections among the downstream targets of miR‐138‐5p predicted on RNA22, TargetScan, picTar and miRDB (left panel) and Venn diagram of the intersections among the overlapped downstream targets of miR‐138‐5p predicted by the above four software, DGEs from TCGA dataset, and the mitochondrial‐related genes from MitoCarta (right panel). DGEs, differentiated expression genes. (B) The expressions of MCU, MOCS1 and LYPLA1 were analysed in GSE174519 dataset. (C) The expressions of MCU, MOCS1 and LYPLA1 were analysed by qPCR in DLD‐1 and Ls174‐T cells, treated as indicated. (D) Western blot analyses for protein expression levels of MCU in DLD‐1 and Ls174‐T cells, treated as indicated. (E) miR‐138‐5p targeted MCU, validated by dual luciferase assay (MCU‐WT, wild‐type MCU containing the binding site; MCU‐MUT, mutant MCU without the binding site). (F) qPCR analysis for expression of MCU in paired tumour (T) and normal (N) tissues (*n* = 30). (G) The expression of miR‐138‐5p and MCU was negatively correlated. *p* values were shown as: *p < 0.05; **p < 0.01; ns, not significant.

Bioinformatics analysis was performed to further explore the role of miR‐138‐5p in CRC. The potential target genes of miR‐138‐5p were sorted out by the prediction software (miRDB, Targetscan, RNA22, and miRWalk). A total of 65 genes were found as the putative target genes of miR‐138‐5p (Figure 4A and Table S4). Furthermore, three genes, including MCU, MOCS1 and LYPLA1, were screened out after overlapping the above 65 genes, the differential expression genes (DEGs) in CRC from TGCA and the mitochondria‐associated genes from MitoCarta. The expressions of MCU and LYPLA1 were significantly upregulated in CRC both in GSE174519 and TCGA datasets (Figures 4B and S3A). In addition, the results from qPCR and western blot indicated that upregulation of miR‐138‐5p notably decreased both the mRNA and protein levels of MCU, whereas downregulation of miR‐138‐5p remarkably increased both the mRNA and protein levels of MCU in vitro and in vivo (Figures 4C,D and S3B,C). Dual luciferase reporter assay was applied to verify the above bioinformatics predictions. Our results showed that the luciferase activity in Ls174T and DLD‐1 cells transfected with miR‐138‐5p mimics and MCU‐WT plasmid was notably attenuated (Figure 4E). However, miR‐138‐5p exhibited no effects on the luciferase activity in Ls174T and DLD‐1cells transfected with MCU‐MUT plasmid (Figure 4E).

The results from qPCR analysis and IHC revealed that MCU expression level was upregulated in CRC (Figures 4F and S3C). Kaplan–Meier analysis presented that CRC patients with high expressions of MCU displayed slower OS compared with those with low expressions of MCU (Figure S3D). Furthermore, the expression of miR‐138‐5p and the mRNA level of MCU showed significantly negative correlation (Figure 4G). Collectively, these results strongly implied that MCU was a potential therapeutic target of miR‐138‐5p in CRC.

### 3.5 miR‐138‐5p targeted MCU to suppress mitochondrial biogenesis in CRC

**FIGURE 5:**
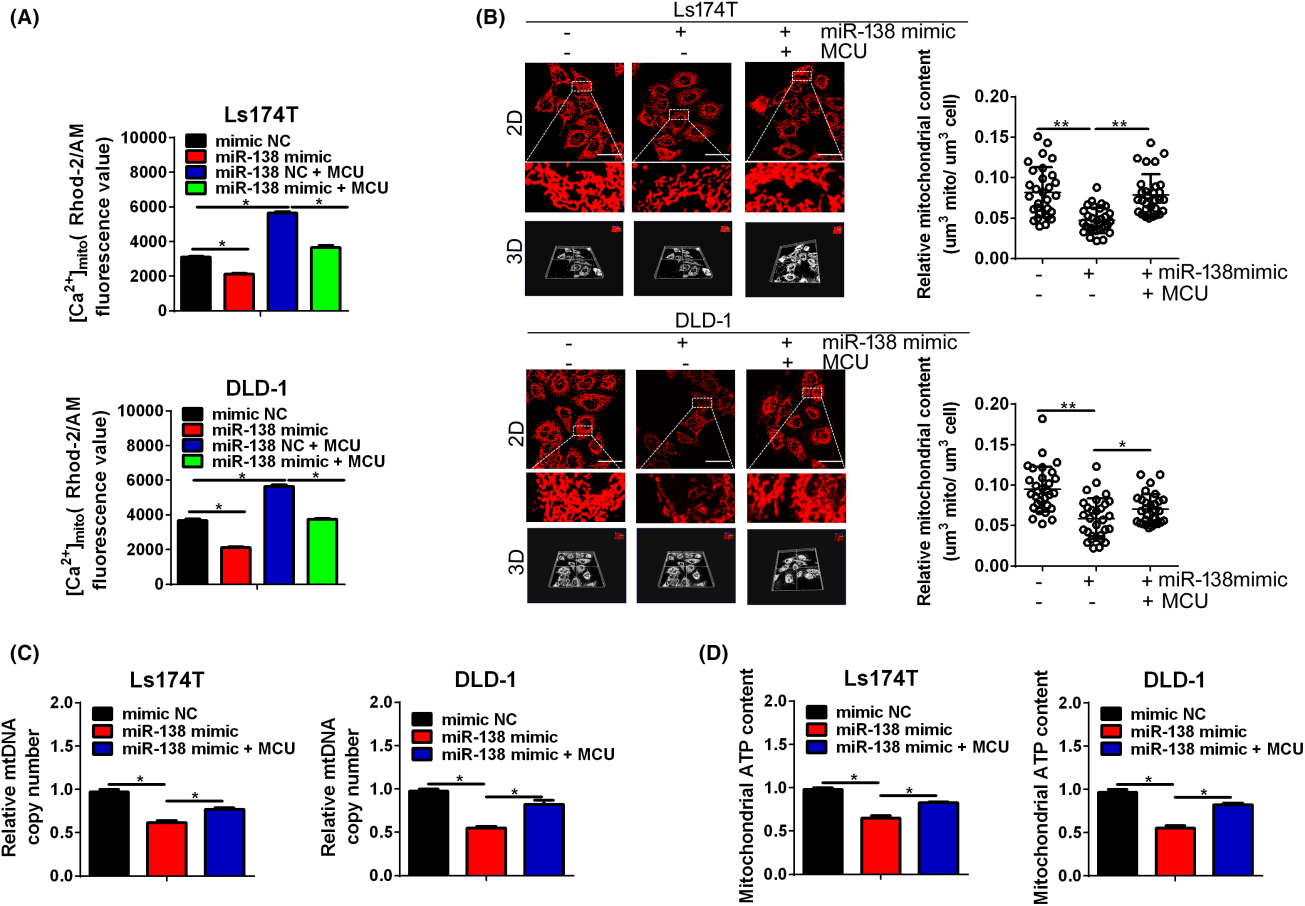
miR‐138‐5p suppressed mitochondrial biogenesis partially through targeting MCU (A) Quantification of [Ca^2+^]_mito_ in Ls174T cells treated as indicated. (B) Relative mitochondrial content (μm^3^ mitochondrial per μm^3^ cell) was determined based on a three‐dimensional model of confocal microscope images in Ls174T cells treated as indicated. Scale bar, 50 μm. (C) Relative mtDNA copy number was determined by qPCR in Ls174T cells, treated as indicated. (D) Mitochondrial ATP levels were determined using the ATP determination kit in Ls174T cells, treated as indicated. *p* values were shown as: *p < 0.05, **p < 0.01.

As the main function of MCU is to uptake calcium to mitochondria, we first explored whether miR‐138‐5p regulated the mitochondrial Ca^2+^ homeostasis via MCU in CRC cells. Our results showed that miR‐138‐5p upregulation reduced in the basal level of mitochondrial Ca^2+^ compared with the control. Moreover, overexpression of MCU could reverse the miR‐138‐5p upregulation‐induced mitochondrial Ca^2+^ reduction in CRC cells (Figures 5A and S4A,B). In conclusion, these results indicated that miR‐138‐5p participates into the regulation of mitochondrial Ca^2+^ homeostasis via MCU.

Some studies have shown that mitochondrial Ca^2+^ homeostasis was closely related with mitochondrial biogenesis in CRC. Therefore, we explored the effects of miR‐138‐5p on the mitochondrial biogenesis in CRC cells. As shown in Figure 5B–D, upregulation of miR‐138‐5p significantly decreased the mitochondrial content, mtDNA copy number, ATP production compared with the corresponding control cells. Moreover, increase in the level of mitochondrial Ca^2+^ by overexpression of MCU reversed the suppression effects of miR‐138‐5p upregulation on mitochondrial biogenesis in CRC cells (Figure 5B–D). Altogether, these results suggest that miR‐138‐5p regulates mitochondrial biogenesis primarily through MCU in CRC cells.

### 3.6 miR‐138‐5p targeted MCU to suppress ROS production in CRC

**FIGURE 6:**
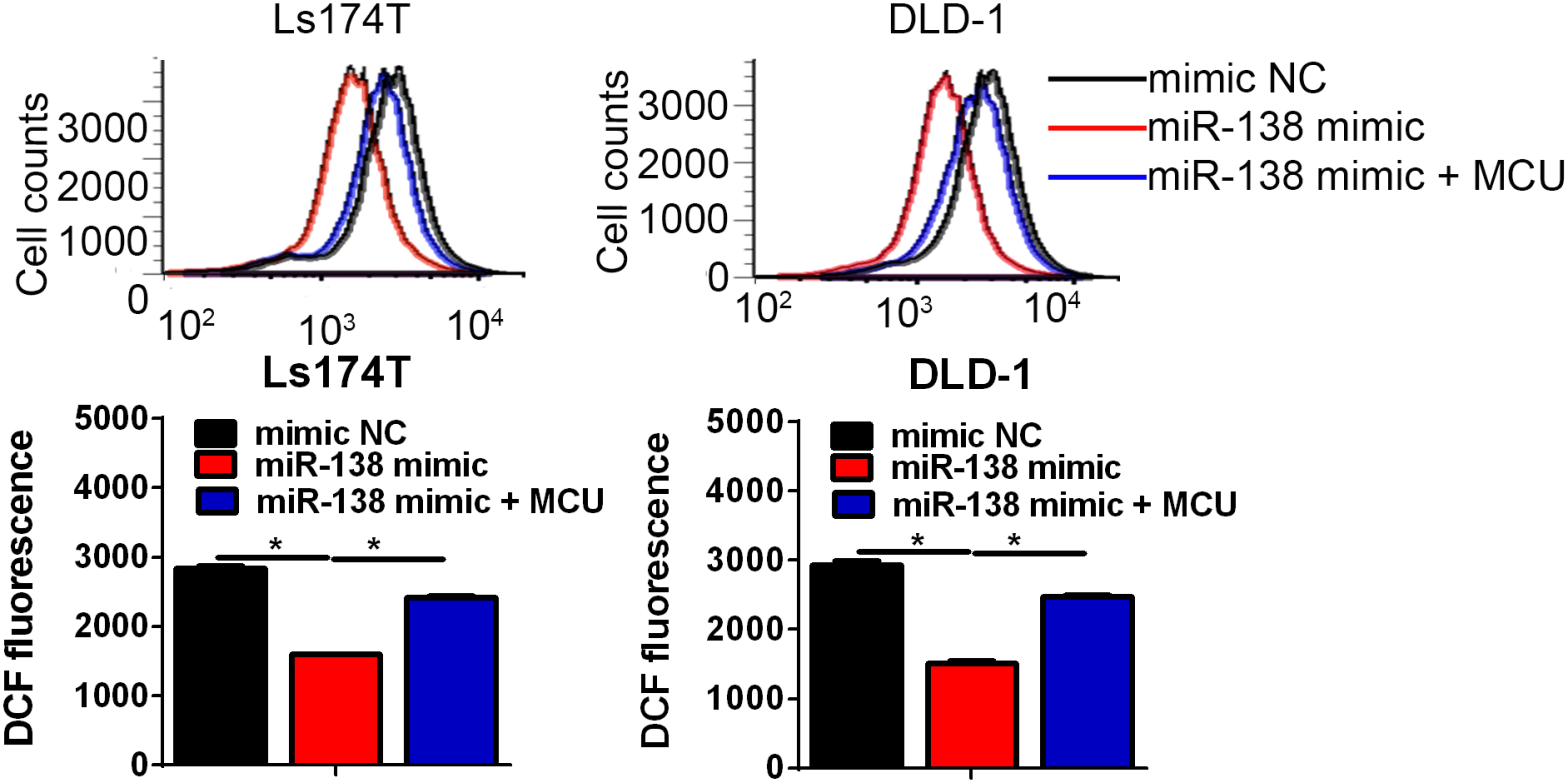
miR‐138‐5p regulated ROS production through MCU in CRC representative confocal microscope images of cellular ROS using DCF in CRC cells, treated as indicated. Data shown are the mean ± SEM from three independent experiments. *p* values were shown as: *p < 0.05.

As previous published studies have demonstrated that mitochondrial Ca^2+^ participates in the process of the ROS production, we determined whether miR‐138‐5p could effect on ROS generation by MCU‐mediated mitochondrial calcium uptake in CRC cells. As shown in Figure 6, upregulation of miR‐138‐5p lowered the total ROS levels in CRC cells compared with the corresponding control cells. Moreover, overexpression of MCU reversed the effects caused by miR‐138‐5p upregulation on reducing the level of total ROS in CRC cells. All in all, these findings suggest that miR‐138‐5p suppressed ROS production via MCU in CRC cells.

### 3.7 miR‐138‐5p suppresses CRC growth via regulating MCU/ROS signalling

**FIGURE 7:**
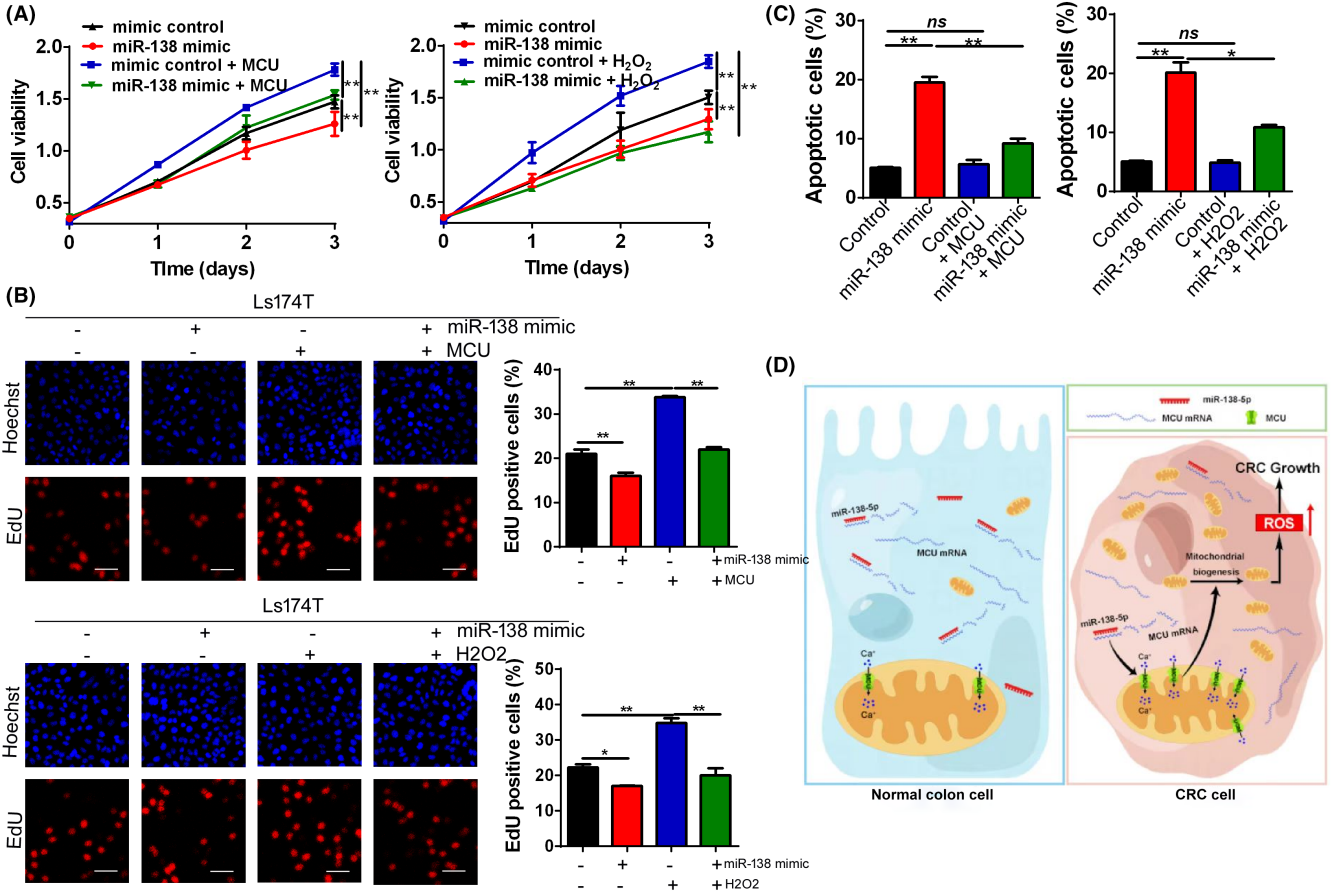
miR‐138‐5p inhibited cell survival of CRC via regulating MCU/ROS signalling (A) CCK‐8 assays of CRC cells, treated as indicated. (B) Representative images of EdU incorporation assay for cell proliferation in CRC cells, treated as indicated. Scale bar, 50 μm. (C) Flow cytometry analysis of cell apoptosis by Annexin‐V/PI staining in CRC cells, treated as indicated. (D) Schematic depicting the regulation of cell survival by miR‐138‐5p in colorectal carcinoma. MCU, mitochondrial calcium uniporter; ROS, reactive oxygen species. *p* values were shown as: *p < 0.05; **p < 0.01; ns, not significant.

The above results indicated that downregulated miR‐138‐5p in CRC promoted tumour growth via MCU/ROS axis. To prove this, H_2_O_2_, a highly ROS activator, was used to increase the ROS level in CRC. The results indicated that MCU overexpression enhanced cell viability and cell proliferation activity, clearly reversed the inhibitory effect of miR‐138‐5p upregulation on cell growth in CRC (Figure 7A,B). Moreover, overexpression of MCU decreased cell apoptosis, and clearly reversed the promoting effect of miR‐138‐5p upregulation on cell apoptosis in CRC (Figures 7C and S5). Furthermore, the similar results were obtained after treatment with H_2_O_2_ in CRC cells (Figures 7A–C and S5). Altogether, these results supported the notion that miR‐138‐5p suppresses the CRC growth via regulating MCU/ROS signalling pathway.

## 4 DISCUSSION

Several studies indicated that miR‐138‐5p was closely related with cancer progression, with entirely different pathogenic mechanisms depending on the type of malignancy. However, the functional roles of miR‐138‐5p in CRC remained unclear. Two major findings were observed in the present study. First, downregulated miR‐138‐5p promotes CRC cell growth via MCU/ROS signalling. Second, downregulated miR‐138‐5p and increased mitochondrial biogenesis are linked together, which provides a novel understanding about the regulation of mitochondrial functions.

Numerous studies have reported that the expression of miR‐138‐5p is reduced in various types of malignancy, including hepatocellular carcinoma,^4^ colorectal carcinoma,^3^, ^5^, ^6^ gastric cancer,^7^ pancreatic cancer,^8^, ^9^ ovarian cancer^10^ and oesophageal squamous cell carcinoma (ESCC).^11^ Consistently, our data indicated that miR‐138‐5p was significantly downregulated in CRC tissues and CRC cell lines, and contributed to tumour progression. Moreover, our results from TCGA‐based prognostic analysis pointed out that downregulated miR‐138‐5p predicted a shorter overall survival in CRC patients. Zheng et al. reported that ESCC patients with lower tissue/serum levels of miR‐138 had lower 5‐year overall survival rate.^11^ You et al. reported that the expression of miR‐138 was related with the differentiation, lymph node metastasis, distant metastasis and TNM stage in colon cancer, and patients with high expression levels of miR‐138 had longer overall survival.^5^ The results from Xu et al. showed that the expression levels f miR‐138‐5p was associated with the lymph node metastasis in CRC.^3^ Altogether, these results indicate that miR‐138‐5p is downregulated in colorectal carcinoma, strongly suggesting that miR‐138‐5p may serve as a promising prognostic biomarker for CRC patients.

Previous literatures reported that miR‐138‐5p functioned distinct roles in different types of malignancy. For instance, Fan et al. found that miR‐138‐5p inhibited cell migration and invasion in cervical cancer.^13^ The results from Xu et al. indicated that miR‐138‐5p regulated cell migration and chemoresistance in colorectal cancer.^3^ As far as we know, the present study might be the first to provide evidence that miR‐138‐5p is crucial for CRC cell growth and mitochondrial biogenesis by MCU/ROS signalling axis. Accumulating evidence indicated that miR‐138‐5p participated into the process of the regulation of cell proliferation in cancers.^5^, ^7^, ^8^, ^12^ You et al. showed that miR‐138 suppressed cell proliferation in colon cancer, suggested that miR‐138 might be involved in the proliferation regulation in colon cancer. The results from Wang et al. indicated that over‐expression of miR‐138 significantly suppressed gastric cancer cell proliferation.^7^ Over‐expression of miR‐138 suppressed cell proliferation in colorectal cancer.^12^ Consistent with the above studies, our results indicated that miR‐138‐5p suppressed cell proliferation in CRC. Several studies have indicated that deregulation miR‐138‐5p is involved in cell death in types of malignancy. miR‐138‐5p inhibitor significantly reversed the promoting effects of TRIM65 knockdown on cisplatin‐induced apoptosis.^14^ A recent study also reported that miR‐138 could sensitize CRC to oxaliplatin to induce cell apoptosis.^15^ Our data indicated that miR‐138‐5p promoted cell apoptosis, whereas downregulation of miR‐138‐5p attenuated 5‐FU‐induced apoptosis in CRC.

The study from Hong et al have indicated that elevated miR‐138 downregulates MCU directly in pulmonary arterial hypertension, and anti‐miRs against miR‐138 restore MCU expression.^16^ Consistently, MCU was predicted as the potential target gene of miR‐138‐5p by bioinformatics methods. Furthermore, MCU was validated as a target of miR‐138‐5p in CRC. However, apart from MCU, accumulating evidence indicated that miR‐138‐5p involved in the regulation of colorectal carcinoma initiation and progression through other targets. For instance, miR‐138‐5p regulated the proliferation, migration and metastasis of CRC through eukaryotic translation initiation factor 4E binding protein 1(4EBP‐1).^17^ Yan et al. found that miR‐138‐5p affected the epithelial‐to‐mesenchymal transition (EMT) of CRC through targeting ZEB2.^18^ Xu et al. reported that miR‐138 regulated CRC cell proliferation, migration, invasion and EMT via podocalyxin‐like protein.^12^ The results from Wang et al. indicated that miR‐138 contributed to the tumourigenesis of CRC through regulating SLC38A1 expression.^19^ Xu et al. reported that miR‐138‐5p suppressed CRC cell migration and chemoresistance through targeting the NFIB‐Snail1 axis.^3^ Wang and colleagues suggested that miR‐138 decreased the oxaliplatin resistance of CRC cells through suppressing PDK1 expression.^15^ The results from Zhao et al. suggested that MIR17HG regulated HK1 expression by sponging miR‐138‐5p, resulting to aggressive invasion and liver metastasis.^20^

Downregulated miR‐138‐5p notably increased mitochondrial biogenesis by elevating MCU‐mediated mitochondrial calcium uptake in CRC. Mitochondrial biogenesis is a basic process required to maintain normal mitochondria function and as an accommodative mechanism in response to changing energy requirements.^21^ Furthermore, is has been recently proven that MCU‐mediated mitochondrial calcium uptake significantly enhanced mitochondrial biogenesis by regulating the TFAM dephosphorylation.^22^ Therefore, we hypothesized that miR‐138‐5p‐associated mitochondrial biogenesis might be related to the MCU‐mediated dephosphorylation of TFAM, which needs to be investigated in the future. Our data clearly demonstrated that miR‐138‐mimics decreased mitochondrial biogenesis, whereas miR‐138‐inhibitor increased mitochondrial biogenesis in CRC. Moreover, MCU overexpression abolished the suppressing effect of miR‐138‐mimic on mitochondrial biogenesis in CRC. Consisted with the previous findings, these results strongly suggested that MCU might be an vital factor in regulating mitochondrial biogenesis.^22^, ^23^ Our results provided elaborate understanding the role of miR‐138‐5p/MCU axis in regulating mitochondrial biogenesis. Moreover, several studies have demonstrated that incorrect mitochondrial biogenesis increased the risk for colorectal cancer.^24^ For instance, Ha et al. reported that proton beam irradiation inhibited metastasis by increasing mitochondrial biogenesis in HT‐29 cells.^25^ Wang et al. demonstrate that mtSSB regulated mitochondrial biogenesis, which was closely associated with the CRC proliferation.^26^ In the present study, our results indicated that downregulated miR‐138‐5p increases mitochondrial biogenesis in CRC.

Reactive oxygen species (ROS) are mainly produced from mitochondria, and their function is critically controlled by calcium during physiological variations of workload.^27^ Growing evidence indicates that accumulation of mitochondrial calcium leads to ROS production.^28^ Ren et al. reported that MCUR1‐mediated mitochondrial calcium accelerated the EMT process by activating ROS/Nrf2/Notch1 pathway in HCC.^28^ Xu et al. indicated that mitochondrial calcium dyshomeostasis led to mitochondrial dysfunction in anaplastic thyroid carcinoma cells, which was closely associated with production of mitochondrial ROS.^29^ In the present study, our results indicated that the miR‐138‐5p overexpression in CRC cells notably reduced mitochondrial calcium uptake and decreased the production of ROS. MCU overexpression remarkably abolished the inhibiting effect of miR‐138‐5p mimic on ROS production, suggested that downregulated miR‐138‐5p increased the production of ROS through MCU in CRC. Very similarly, Wang et al. reported that the expression of miR‐138 was closely associated with the generation of ROS in HT29/R and SW480/R.^15^ As far as we all know, the present study might provide the first mechanistic insights into miR‐138‐5p/MCU axis‐mediated mitochondrial biogenesis in CRC.

A changing redox status accompanied by increased production of ROS has been implicated in various of diseases including CRC.^30^ Growing evidence indicated that ROS participated into the activation process of phosphatidylinositol 3‐kinase‐protein kinase B (PI3K)–Akt signalling and the mitogen‐activated protein (MAP) kinase signalling, thus contributing to aggressive cell proliferation, and migration in cancers.^30^ Ren et al. found that increased ROS accelerated cell invasion and migration via activating JNK pathway in HCC.^31^ Liu and colleagues suggested that ROS promoted CRC cell growth via activating NFκB pathway.^22^ In the present study, our data revealed that downregulated miR‐138‐5p promoted ROS production via increasing the expression of MCU, and increased MCU and ROS production involved into the miR‐158‐5p‐mediated aggressive growth in CRC, but the downstream signal pathway warranted systematic investigation in the future.

In conclusion, we thoroughly investigated the function of downregulated miR‐138‐5p in accelerating CRC cell growth both in vitro and in vivo. Our results clearly indicated that downregulated miR‐138‐5p‐mediated MCU upregulation notably increased mitochondrial biogenesis. Moreover, we also clarified that downregulated miR‐138‐5p promoted CRC growth by enhancing ROS production. Our findings uncover a novel mechanism underlying miR‐138‐5p/MCU axis in facilitating CRC cell growth. However, the underlying mechanism downregulated miR‐138‐5p‐mediated upregulation of MCU promoted mitochondrial biogenesis was unclear, which needs to be clarified in the future.

## AUTHOR CONTRIBUTIONS

**Jianjun Zhu:** Conceptualization (lead); data curation (equal); formal analysis (equal); funding acquisition (lead); investigation (lead); methodology (lead); project administration (equal); resources (supporting); software (supporting); validation (lead); visualization (lead); writing – original draft (lead). **Chunle Zhang:** Conceptualization (lead); data curation (equal); formal analysis (equal); project administration (equal); resources (equal); supervision (equal); visualization (equal); writing – review and editing (supporting). **Zhengjie Wang:** Investigation (supporting); methodology (supporting); software (supporting); validation (supporting). **Lihong Shi:** Investigation (supporting); methodology (supporting); software (supporting); validation (supporting); visualization (supporting). **Li Li:** Data curation (supporting); formal analysis (supporting); investigation (supporting); methodology (supporting); project administration (equal); software (supporting); supervision (equal); validation (supporting). **Hao Wu:** Conceptualization (supporting); formal analysis (supporting); funding acquisition (equal); methodology (equal); software (equal); validation (equal); visualization (equal); writing – original draft (equal). **Ming Liu:** Conceptualization (equal); data curation (equal); funding acquisition (equal); investigation (lead); project administration (equal); resources (equal); supervision (lead); visualization (supporting); writing – review and editing (lead).

## CONFLICT OF INTEREST STATEMENT

The authors declare that they have no competing interests.

## Supporting information


DataS1

## Data Availability

All data generated or analyzed during the present study are available from the corresponding author.
