# Supplementary material for: miR‐138‐5p targets MCU to inhibit mitochondrial biogenesis and colorectal cancer growth
Source: J Cell Mol Med. 2023 Jun 1;27(15):2112–22. doi: 10.1111/jcmm.17798 (PMC10399525; doi:10.1111/jcmm.17798)
Supplement: Supplementary file 1 — DataS1 [file JCMM-27-2112-s001.docx]

**MiR-138-5p targets MCU to inhibit mitochondrial biogenesis and colorectal cancer growth**

Jianjun Zhu^1#^, Chunle Zhang^2#^, Zhengjie Wang^3^, Lihong Shi^4^, Li Li^1^, Hao Wu^1*^, Ming Liu^1*^

^1^Department of Medical Cellular Biology and Genetics, Shanxi Medical University, Taiyuan, China

^2^Department of Nephrology, Kidney Research Institute, West China Hospital of Sichuan University, Chengdu 610041, China

^3^Department of Nuclear Medicine, The First Affiliated Hospital of Chongqing Medical University, Chongqing, China

^4^Department of Human Anatomy, Shanxi Medical University, Taiyuan, China

#Jianjun Zhu and Chunle Zhang contributed equally to this work

**Supplementary Materials and Methods**

**Microarray analysis**

All public mRNA microarray datasets (GSE174519) and miRNA microarray datasets (GSE73487, GSE147603, and GSE126093) used in this study were downloaded from the Gene Expression Omnibus (GEO) in the NCBI web server. Expression level of per gene was calculated from Log2 of its upper quartile FPKM (FPKM-UQ) value. Differentially expressed miRNAs/genes were analyzed with Log_2_FC>1.0 and *P*<0.05 as the criteria. The RNA22 database (<http://cm.jefferso.edu/rna22>), miRDB (<http://www.mirdb.org>), miRWalk (<http://mirwalk.umm.uni-heidelberg.de/>), and TargetScan (<http://www.targetscan.org/mamm>) database were applied to predict the binding sites between miRNAs and target genes. The prediction outcomes were integrated and analyzed using a Venn diagram produced by online tools ( <http://bioinformatics>.psb.ugent.be/webtools/Venn/).

**Cell culture**

Normal human colorectal cell line FHC and 6 colorectal carcinoma cell lines Lovo, Ls174T, SW480, SW620, HCT-116, and DLD-1(ATCC, Manassas, USA) were incubated in DMEM (Gibco, NY, USA) supplemented with 10% fetal bovine serum (FBS), penicillin (100U/mL) and streptomycin (100 mg/mL)(37℃ with 5% CO_2_)。

**RNA extraction and Quantitative Polymerase Chain Reaction (qPCR)**

Total RNA was isolated from CRC cells and tumor tissues using TRIzol reagent (Invitrogen, USA) following the manufacturer’s instructions. Reverse transcription was performed sing a PrimeScript RT reagent kit (Takara, China) or miRNA First Strand cDNA Synthesis (Sangon △Biotech, China ) to produce cDNA. qPCR was performed using the SYBR Green PCR kit (Takara, China). U6 was used as the internal control for miR-138-5p, while β-actin for protein-coding genes. Relative gene expression was calculated using the 2^-△Ct^ method. The primer sequences used in the present study were shown in Table S2.

**Western blot assay**

Western blot assay was performed as previously described. Briefly, protein samples loaded into the 10% PAGE were separated and transferred to PVDF membrane (Invitrogen). After blocking the PVDF membrane with TBST buffer containing 5% nonfat-milk powder, the PVDF membrane was incubated with the primary antibody at 4°C overnight, followed by incubating with the secondary antibody (1:10,000) at room temperature for 2h. In the end, the protein bands were quantified by Quantity One Software. Antibodies used in this study were listed in Table S3. **TUNEL assay**

The apoptosis in the xenograft tissues was evaluated using the terminal deoxynucleotidyl transferase-mediated dUTP nick-end labeling (TUNEL) kit (Roche Applied Science, Rotkreuz, Swtitzerland) according to the manufacturer’s instructions. Briefly, tissue sections were incubated with the Proteinase K at 37℃for 30 min, followed by incubating with TUNEL reaction buffer at 37℃ for 1 hour protected from light and stained with DAPI. Images of the tissue sections were obtained using the laser confocal microscopy.

**Supplemental Table 1. Sequences of miRNA mimics and miRNA inhibitors.**

| **1. miRNA mimics and inhibitor(5′-3′)** | | |
| --- | --- | --- |
| MiR-138 mimics | sense | AGCUGGUGUUGUGAAUCAGGCCCG |
|  | Anti-sense | AGCUGGUGUUGUGAAUCAGGCCCG |
| MiR-138 inhibitors | CGGCCUGAUUCACAACACCAGCU | |

**Supplemental Table 2. Sequence of primers**

| **1. Primers used in q-PCR analysis** | | |
| --- | --- | --- |
| MCU | forward primer | TCCAGAAGCCAGAGACAGAC |
|  | reverse primer | TGTCGGAGAGGCAGATGTAC |
| MOCS1 | forward primer | TGGATGTGCGCTTCATAGAG |
|  | reverse primer | GTTGCAGGTCCCACAGAAAT |
| LYPLA1 | forward primer | GTGGCTATGCCTTCATGGTT |
|  | reverse primer | CACCGATAGGACCCTGAGAA |
| β-actin | forward primer | ACTCTTCCAGCCTTCCTTCC |
|  | reverse primer | TCTCCTTCTGCATCCTGTCG |
| ND1 | forward primer | ATGGCCAACCTCCTACTCCT |
|  | reverse primer | GCGGTGATGTAGAGGGTGAT |
| HGB | forward primer | GCTTCT GACACAACTGTGTTCACTAGC |
|  | reverse primer | CACCAACTTCATCCACGTTCACC |
|  |  |  |
|  |  |  |
|  |  |  |
|  |  |  |
| **2. Primers used in q-PCR analysis for miRNAs(5′-3′)** | | |
| MiR-138-5p | Forward primer | AGCTGGTGTTGTGAATCAGGCC |
|  |  |  |
|  |  |  |
|  |  |  |

**Supplemental Table 3. Primary antibodies used for Western blotting.**

| **Antibody** | **Company (Cat.NO.)** | **Working dilutions** |
| --- | --- | --- |
| MCU | SIGMA (HPA05189) | WB: 1/200 |
| β–actin | Proteintech (20536-1-AP) | WB: 1/2000 |

**Supplemental Table 4. Target gene list of miR-138-5p**

| Gene | Full name | Other names |
| --- | --- | --- |
| RARA | retinoic acid receptor alpha | NR1B1, RAR, RARalpha |
| BNIP3L | BCL2 interacting protein 3 like | BCL2 interacting protein 3 like |
| SIN3A | SIN3 transcription regulator family member A | WITKOS |
| JAZF1 | JAZF zinc finger 1 | TIP27, ZNF802 |
| PDE3A | phosphodiesterase 3A | CGI-PDE, CGI-PDE A, CGI-PDE-A, HTNB |
| RHOC | ras homolog family member C | ARH9, ARHC, H9, RHOH9 |
| TP53INP2 | tumor protein p53 inducible nuclear protein 2 | C20orf110, DOR, PIG-U, PIGU, PINH, dJ1181N3.1 |
| CLNS1A | chloride nucleotide-sensitive channel 1A | CLCI, CLNS1B, ICln |
| FEM1C | fem-1 homolog C | EUROIMAGE686608, EUROIMAGE783647, FEM1A |
| LYPLA1 | lysophospholipase 1 | APT-1, APT1, LPL-I, LPL1, hAPT1 |
| TIPARP | TCDD inducible poly(ADP-ribose) polymerase | ARTD14, PARP7, pART14 |
| ARRDC3 | arrestin domain containing 3 | TLIMP |
| SOX12 | SRY-box transcription factor 12 | SOX22 |
| SNCB | synuclein beta |  |
| CALN1 | calneuron 1 | CABP8 |
| SLC17A7 | solute carrier family 17 member 7 | BNPI, VGLUT1 |
| PDE7B | phosphodiesterase 7B | bA472E5.1 |
| ZNF444 | zinc finger protein 444 | EZF-2, EZF2, ZSCAN17 |
| MCU | mitochondrial calcium uniporter | C10orf42, CCDC109A, HsMCU |
| USP10 | ubiquitin specific peptidase 10 | UBPO |
| SIRT1 | sirtuin 1 | SIR2, SIR2L1, SIR2alpha |
| THAP11 | THAP domain containing 11 | CTG-B43a,CTG-B45d, HRIHFB2206, RONIN |
| MACF1 | microtubule actin crosslinking factor 1 | ABP620, ACF7, KIAA0754, LIS9, Lnc-PMIF, MACF, OFC4 |
| MAP3K11 | mitogen-activated protein kinase kinase kinase 11 | MEKK11, MLK-3, MLK3, PTK1, SPRK |
| EPHA8 | EPH receptor A8 | EEK, EK3, HEK |
| ANK1 | ankyrin 1 | ANK, SPH1, SPH2 |
| EZH2 | enhancer of zeste 2 polycomb repressive complex 2 subunit | ENX-1, ENX1, EZH2b, KMT6, KMT6A, WVS, WVS2 |
| SOX4 | SRY-box transcription factor 4 | CSS10, EVI16 |
| C6orf47 | chromosome 6 open reading frame 47 | D6S53E, G4, NG34 |
| PPARGC1A | PPARG coactivator 1 alpha | LEM6, PGC-1(alpha), PGC-1alpha, PGC-1v, PGC1, PGC1A, PPARGC1 |
| CNOT8 | CCR4-NOT transcription complex subunit 8 | CAF1, CALIF, Caf1b, POP2, hCAF1 |
| KLF12 | Kruppel like factor 12 | AP-2rep, AP2REP, HSPC122 |
| PTP4A1 | protein tyrosine phosphatase 4A1 | HH72, PRL-1, PRL1, PTP(CAAX1), PTPCAAX1 |
| MOCS1 | molybdenum cofactor synthesis 1 | molybdenum cofactor synthesis 1 |
| EFNB3 | EFNB3 | EFL6, EPLG8, LERK8 |
| DVL2 | dishevelled segment polarity protein 2 |  |
| USP47 | ubiquitin specific peptidase 47 | TRFP |
| PTK2 | protein tyrosine kinase 2 | FADK, FADK 1, FAK, FAK1, FRNK, PPP1R71, p125FAK, pp125FAK |
| RAVER1 | ribonucleoprotein, PTB binding 1 |  |
| RELN | reelin | ETL7, LIS2, PRO1598, RL |
| GNAI2 | G protein subunit alpha i2 | GIP, GNAI2B, HG1C, H_LUCA15.1, H_LUCA16.1 |
| RPS6KA1 | ribosomal protein S6 kinase A1 | HU-1, MAPKAPK1, MAPKAPK1A, RSK, RSK1, p90Rsk |
| PSFM1 |  |  |
| ATP11C | ATPase phospholipid transporting 11C | ATPIG, ATPIQ, HACXL |
| ROCK2 | Rho associated coiled-coil containing protein kinase 2 | ROCK-II |
| ZMYND11 | zinc finger MYND-type containing 11 | BRAM1, BS69, MRD30 |
| MGAT5B | alpha-1,6-mannosylglycoprotein 6-beta-N-acetylglucosaminyltransferase B | GnT-IX, GnT-VB |
| SFXN2 | sideroflexin 2 | SLC56A2 |
| LHFPL3 | LHFPL tetraspan subfamily member 3 | LHFPL4 |
| PHOX2B | paired like homeobox 2B | CCHS, NBLST2, NBPhox, PMX2B |
| NEBL | nebulette | C10orf113, LASP2, LNEBL, bA165O3.1 |
| UNC5D | unc-5 netrin receptor D | PRO34692, Unc5h4 |
| MAP2K7 | mitogen-activated protein kinase kinase 7 | JNKK2, MAPKK7, MEK, MEK 7, MKK7, PRKMK7, SAPKK-4, SAPKK4 |
| HIF1AN | hypoxia inducible factor 1 subunit alpha inhibitor | FIH1 |
| SLC20A1 | solute carrier family 20 member 1 | GLVR1, Glvr-1, PIT1, PiT-1 |
| NBEA | neurobeachin | BCL8B, LYST2, NEDEGE |
| TULP4 | TUB like protein 4 | TUSP |
| DHDDS | dehydrodolichyl diphosphate synthase subunit | CIT, CPT, DEDSM, DS, HDS, RP59, hCIT |
| PAPPA | pappalysin 1 | ASBABP2, DIPLA1, IGFBP-4ase, PAPA, PAPP-A, PAPPA1 |
| TRPS1 | transcriptional repressor GATA binding 1 | GC79, LGCR |
| GIT1 | GIT ArfGAP 1 | p95-APP1 |
| RIMS3 | regulating synaptic membrane exocytosis 3 | NIM3, RIM 3, RIM3 |
| MXD4 | MAX dimerization protein 4 | MAD4, MST149, MSTP149, bHLHc12 |

**Supplementary Figure and Figure legend**

**
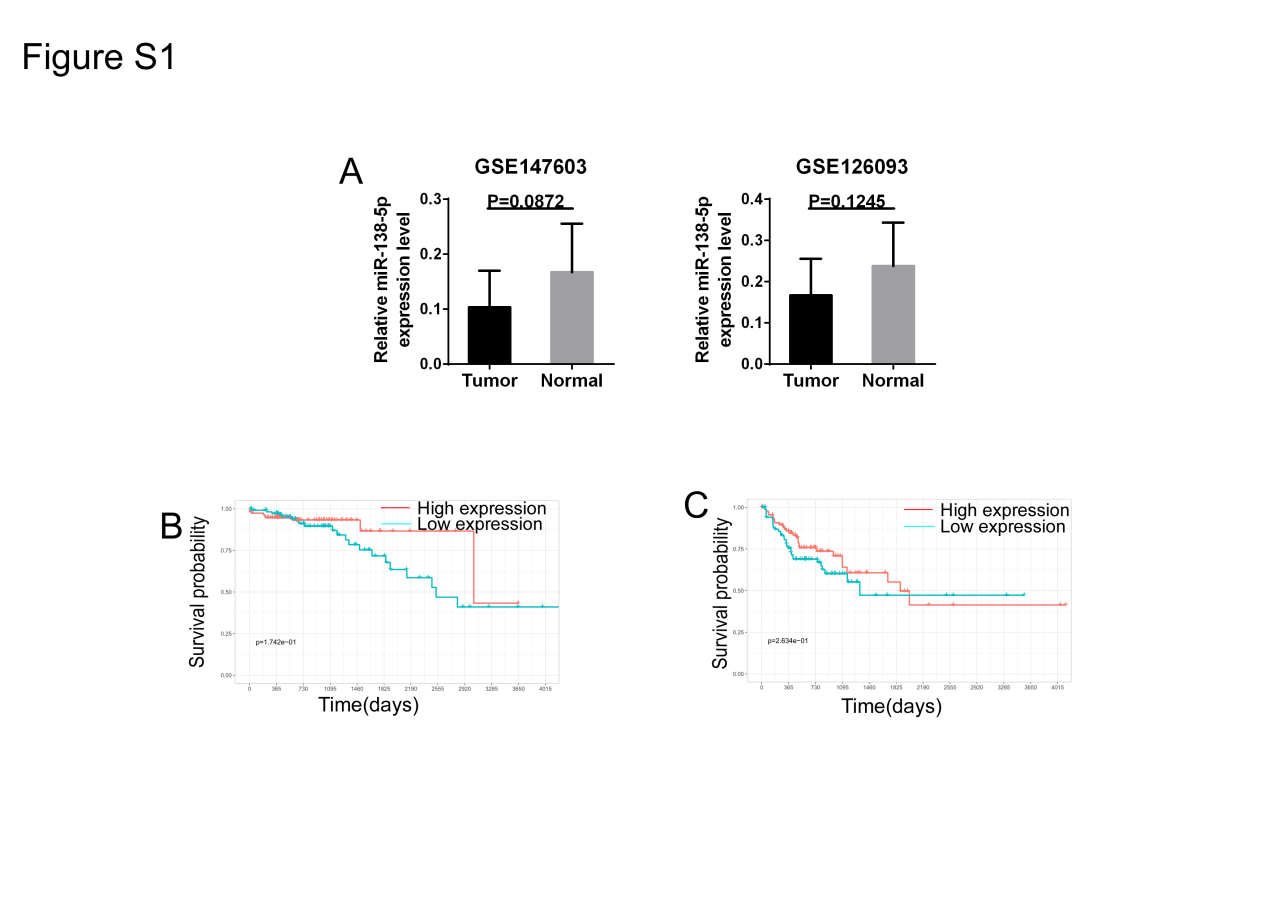
**

**Fig.S1 A** Bioinformatics analysis for miR-138-5p expression based on the GSE147603 and GSE126093 datasets. **B** Kaplan-Meier survival curves for overall survival (OS) stratified by miR-138-5p expression in CRC patients with early stage. **C** Kaplan-Meier survival curves for overall survival (OS) stratified by miR-138-5p expression in CRC patients with advanced stage. **P*<0.05


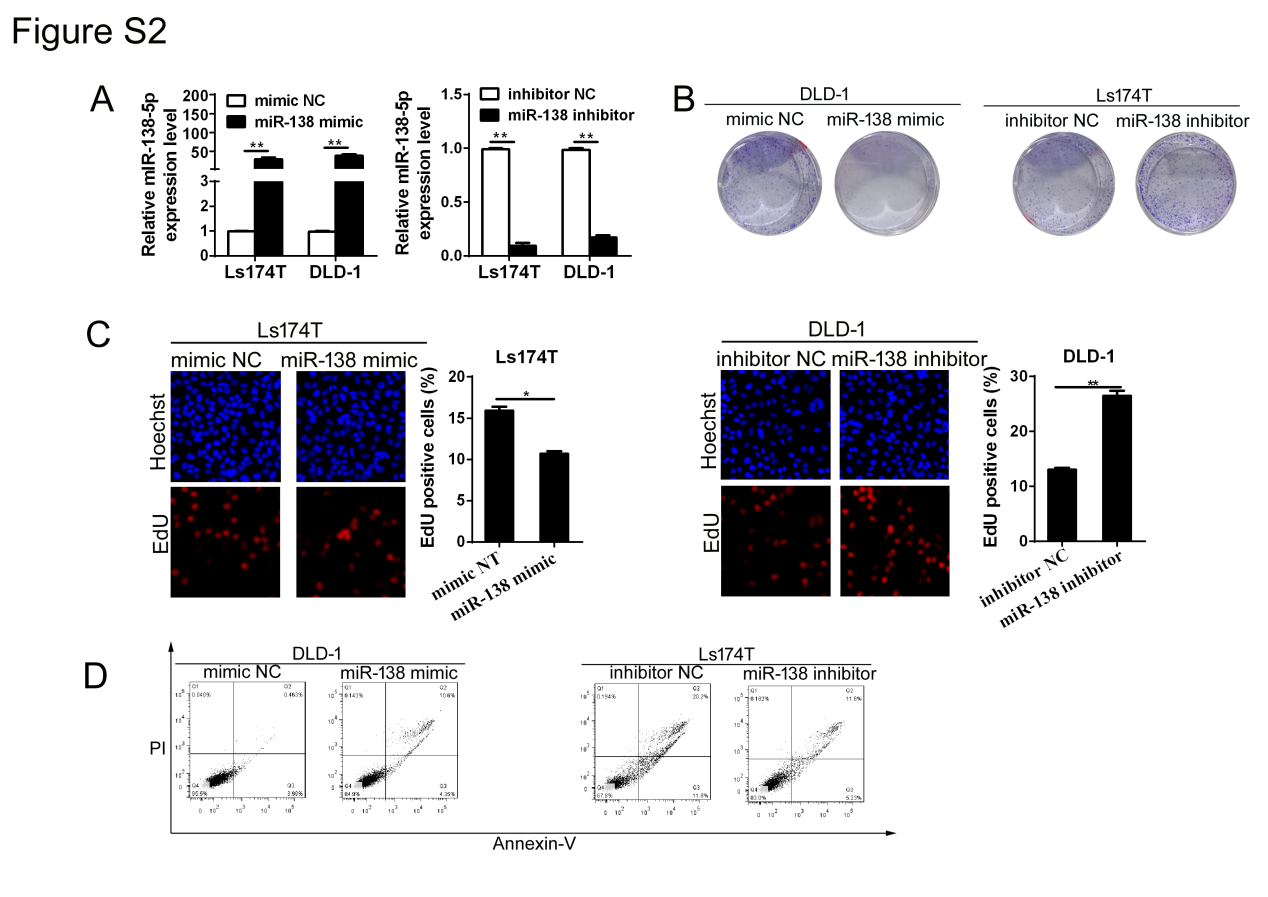


**Fig.S2 A** The expressions of miR-138-5p was analyzed by qPCR in CRC cells, treated as indicated. **B** Colony formation assay in CRC cells treated as indicated. **C** Cell proliferation ability was evaluated using ethynyl deoxyuridine (EdU) incorporation assay 48 h after transfection with treatment as indicated. Scale bar, 50 μm．**D** Flow cytometry analysis of cell apoptosis by Annexin-Ⅴ/PI staining in CRC cells, treated as indicated. Data are presented as mean ± SEM from three independent experiments. **P*<0.05. ***P*<0.01


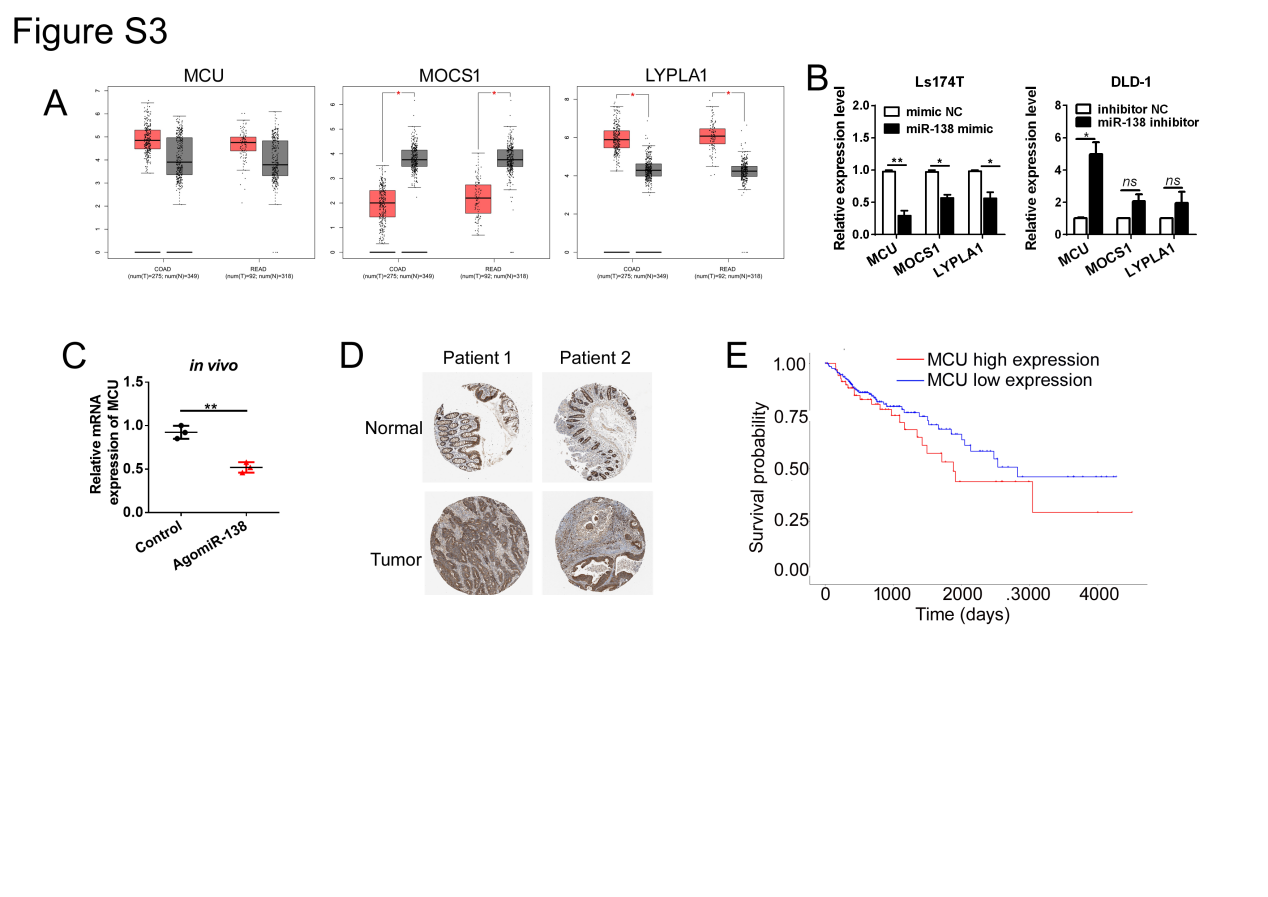


**Fig.S3 A** Bioinformatics analysis for the expression levels of MCU, MOCS1, and LYPLA1 based on TCGA datasets. **B** The expressions of MCU, MOCS1, and LYPLA1 were analyzed by qPCR in DLD-1 cells, treated as indicated. **C** Representative immunohistochemical (IHC) staining images of MCU in normal colorectal tissues and colorectal carcinoma tissues from HPA. **D** Kaplan-Meier survival curves for overall survival (OS) stratified by MCU expression in CRC patients. Data are presented as mean ± SEM from three independent experiments. **P*<0.05; ***P*<0.01


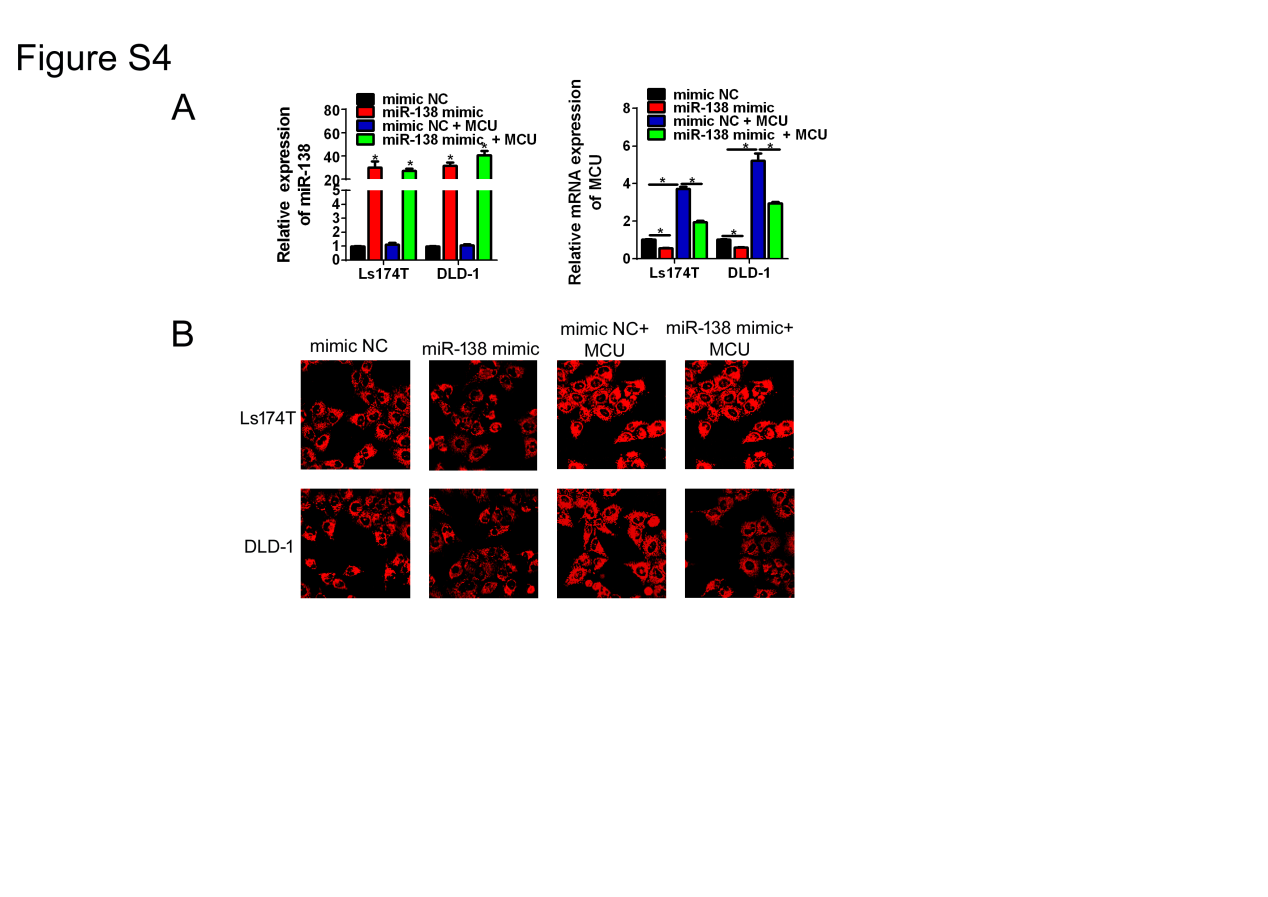


**Fig.S4 A** The expressions of miR-138-5p and MCU was analyzed by qPCR in CRC cells, treated as indicated. **B** Representative confocal microscope images of [Ca^2+^]mito using Rhod-2/AM in Ls174T cells, treated as indicated. Scale bar, 20 μm. Data are presented as mean ± SEM from three independent experiment. *ns*, no significant; **P*<0.05; ***P*<0.01


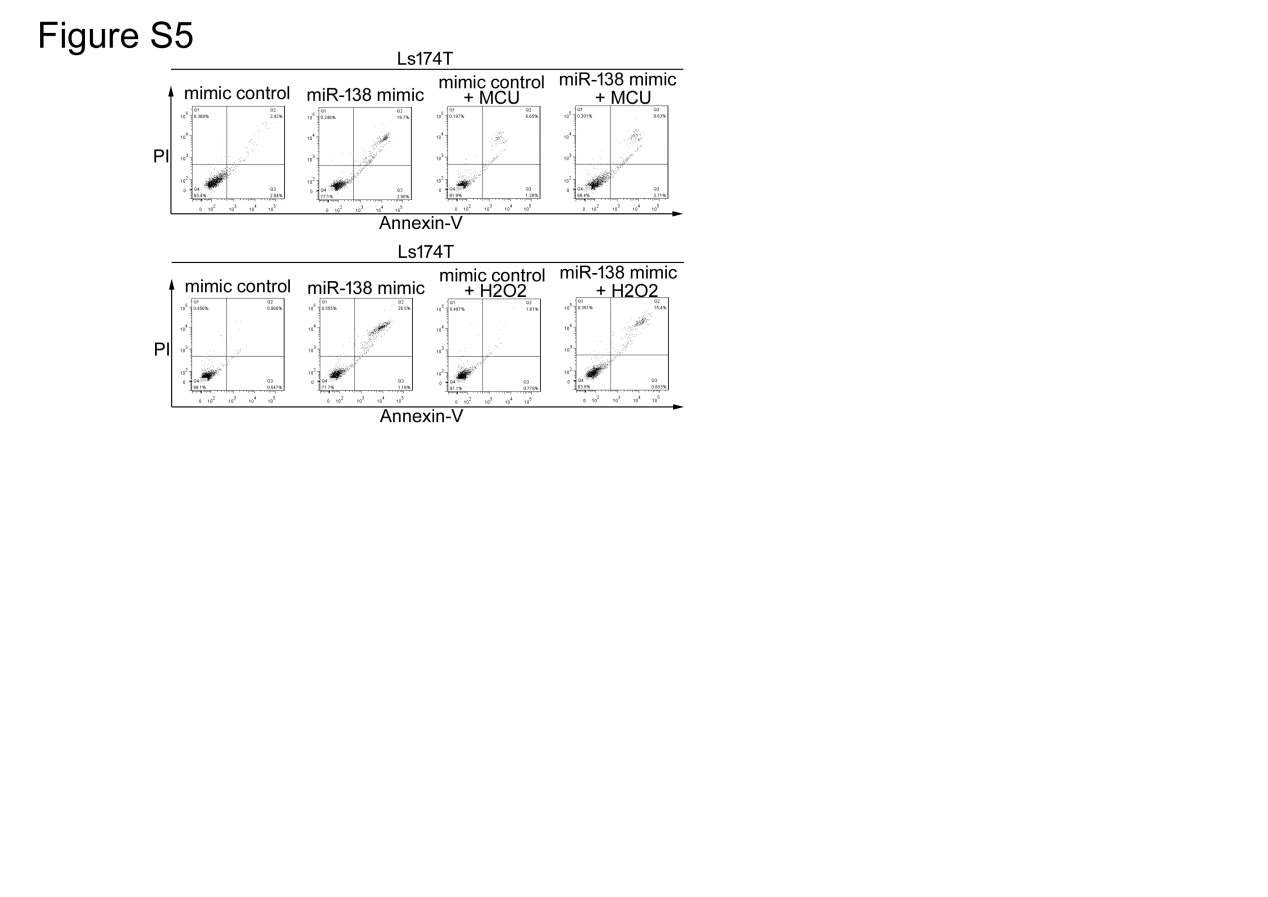


**Fig.S5** Flow cytometry analysis of cell apoptosis by Annexin-Ⅴ/PI staining in CRC cells, treated as indicated.
